# The Institutional Worker

**Published:** 1912-03-09

**Authors:** 


THE
### INSTITUTIONAL WORKER
**Being a Special Supplement to "The Hospital"**

### Editor's Notices.

**Contributions:**

Contributions should be written, or preferably typed, on one side of the paper only, and all articles sent in are accepted upon the distinct understanding that they are forwarded to The Hospital only.

The Editor cannot undertake to return MSS. not used, but when a stamped directed envelope is enclosed the MSS. may be returned if a special request is made.

Accepted articles and paragraphs of news will be paid for after publication at the scale rate.

**Address:**
To prevent delay all contributions and letters on editorial business must be addressed exclusively to the Editor, "The Hospital," 29 Southampton Street, Strand, London, W.C. It is important that this regulation shall be strictly observed.

**Correspondence:**
Correspondence on all subjects is invited. The name and address of correspondents must be given as a guarantee of good faith, but not necessarily for publication.

**Special Articles:**
Special articles are invited, and questions, inquiries, and paragraphs upon all matters relating to the work, administration, and management of general, special, mental, fever, cottage and convalescent hospitals, sanatoria, homes, institutions, societies and organisations for the treatment or care of the sick, injured and dependents of all classes. Every matter affecting the interests and welfare of the staff of all grades working in these institutions will receive special consideration.

**Administrative Medicine, Research and Items of News:**
Special terms will be paid for approved articles on subjects in this wide field by experts who have made some section of it their special study and interest. We wish to give prominence to every movement tending to advance accuracy of diagnosis, efficiency in treatment, and the development of modern methods to eradicate disease and promote the welfare of the suffering.

**A Bureau of Information:**
We invite inquiries and applications for information and help in providing for that numerous class of sufferers who require special care or house-room which they cannot obtain in their own homes or secure for themselves. We cannot, however, prescribe or recommend practitioners.

**Books for Review:**
Publishers are particularly requested to send advance proofs of any new books of importance whenever possible, as the Editor has made arrangements to publish immediate reviews on a new plan.

**Photographs, Plans, Blocks, and Illustrations:**
It is requested that wherever possible MSS. may be accompanied by illustrations in any of the above forms. The name of the sender and of the article to which it belongs should be written on the back of each photograph, drawing, or block for purposes of identification.

**Local Papers:**
Newspapers containing reports or news paragraphs should be marked and addressed to the Sub-Editor.

**The Coupon System:**
A coupon will be found at the bottom of the third inside page of the cover each week. This coupon must be attached to every question or inquiry to which an answer is desired in The Hospital.

### The Institutional Worker.

**Its Object and Aim.**

The term Institutional Worker is employed in its widest sense, and includes every individual engaged in, or connected with Charitable, Poor Law, and Municipal Administration, as well as the Medical, Health, and Sanitary Services. This section is primarily intended to be of assistance to those seeking appointments, and a large number of announcements of vacancies appear in its columns every week. If there is no appointment suited to your especial requirements an advertisement of 21 words, costing 1s., under the heading "Appointments Wanted" will bring your needs prominently before the Managerial Officials of Institutions of every description throughout the United Kingdom.

It is our aim to make The Institutional Worker of the greatest practical utility to everyone engaged in, or attracted to, institutional work, and in this our readers may help us, and themselves, by showing a lively interest in those advertisements which appear from week to week. In this way only can the usefulness of The Institutional Worker be increased and our aim accomplished.

### Manager's Notices.

**Letters relating to the publication, sale, and advertising departments of "The Hospital," must be addressed to The Manager, "The Hospital," The Hospital Building, 28 and 29 Southampton Street, London, W.C.**

**Subscriptions** which may begin at any time, are payable in advance. Cheques and Post-Office orders, crossed London County and Westminster Bank, Covent Garden branch, should be made payable to the Manager of The Hospital.

Rates of Subscription (payable in advance).

| | |
|---|---|
| UNITED KINGDOM. | |
| Three Months (including Postage) ... ... ... ... | 2s. 0d. |
| Six Months do. ... ... ... ... | 4s. 0d. |
| Twelve Months do. ... ... ... ... | 6s. 6d. |
| FOREIGN AND COLONIAL. | |
| Three Months (including Postage) ... ... ... ... | 2s. 6d. |
| Six Months do. ... ... ... ... | 5s. 0d. |
| Twelve Months do. ... ... ... ... | 8s. 8d. |

**Advertisements:**
All advertisements for The Hospital must reach the Manager not later than Wednesday morning in each week. For scale of charges see pages vii and 2.

**Special Rates** will be quoted for those institutions which agree to send all their vacancy, contract, and public notices to The Hospital each year, so as to make it a file of such announcements for ready reference.

This form may be used for small prepaid advertisements, and when filled up should be posted to the

*Manager*, The Hospital,
28 and 29 Southampton Street,
Strand, W.C.

Appointments Wanted, 21 words ... 1s. 0d.
Appointments Vacant, 21 words ... 2s. 6d.

| | | |
|---|---|---|
| | | |
| | | |
| | | |
| | | |
| | | |
| | | |
| | | |

### OFFICIAL APPOINTMENTS VACANT.

### Poor-Law Vacancies.

Assistant Clerk (£250), Carlisle Union and R.D.C. Mr. H. B. Lonsdale, C. to G., Carlisle. March 11.

Assistant Clerk (£70), Bermondsey Guardians. Mr. E. Pitts Fenton, C. to G., 283 Tooley Street, S.E. March 18.

Assistant Male Observation Ward Attendant (20s. weekly), West Ham Union. Mr. T. Smith, C. to G., Leytonstone, N.E. March 13.

Assistant Nurse (£26), Walsingham Union. Mr. R. S. Butcher, C. to G., Fakenham, Norfolk. March 9.

Assistant Nurse (£25), Newmarket Union. Mr. S. J. Ennion, C. to G., Newmarket. March 9.

Assistant Nurse (£25), Amersham Union. Mr. R. H. Rushforth, C. to G., Amersham. March 11.

Assistant Nurse (£22), Bermondsey Guardians. Matron of the Schools, Wickham Road, Shirley, Croydon.

Assistant Nurse (£25), St. Ives (Hunts) Union. Mr. G. D. Day, C. to G., St. Ives, Hunts. March 11.

Assistant Nurse (£25), Luton Union. Mrs. Richmond, Matron, Workhouse Infirmary, Luton. March 12.

Assistant Nurse (£18), Blofield Union. H. H. Cole, C. to G., 12 Bank Street, Norwich. March 15

Assistant Nurse (£28), Newton Abbot Union. F. Horner, C. to G., Union Offices, Newton Abbot. March 19.

Assistant Nurse (£28), Thornbury Union. H. P. Thurston, C. to G., Union Offices, Thornbury, Glos. March 14.

Assistant Nurses (£22 10s.), Watford Union. Mr. F. Wilson, C. to G., Watford. March 12.

Assistant Nursery Attendant (£14), Barnet Union. Matron of the Workhouse, Barnet. March 9.

Barber and Bathman (£25), Cheltenham Union. Mr. J. Meek, C. to G., Cheltenham. March 13.

Boys' Attendant (£30), Barnet Union. Master of the Workhouse, Barnet. March 9.

Charge Nurse (£30), Bristol Guardians. Mr. J. J. Simpson, C. to G., Bristol. March 11.

Charge Nurse (£32), Bedford Union. Mr. W. Payne, C. to G., Bedford. March 18.

Charge Nurse (£30), Scarborough Union. J. W. Read, C. to G., Scarborough. March 20.

Charge Nurse (£35), Bromley Union. E. Haslehurst, C. to G., Union Offices, Park House, Bromley, Kent. March 18.

Children's Attendant (£17), Farnham Union. Miss Tisdell, Superintendent Nurse, The Infirmary, Farnham.

Cook (£22), Lutterworth Union. Mr. T. C. Bodycote, C. to G., Lutterworth. March 20.

Cook-Housekeeper (£30), Coventry Union. Mr. E. A. Evans, C. to G., Coventry. March 14.

Day Charge Nurse (£30), West Bromwich Union. Mr. A. H. Ward, C. to G., West Bromwich. March 14.

Female Imbecile Attendant (£20), Bradford-on-Avon Union. Mr. J. Compton, C. to G., Bradford-on-Avon. March 12.

Foster-Mother (£20), Chapel-en-le-Frith Union. Mr. J. B. Boycott, C. to G., Chapel-en-le-Frith. March 9.

Labour Mistress, etc. (£25), Lewisham Union. Mr. H. C. Mott, C. to G., 394 High Street, Lewisham, S.E. March 16.

Laundress (£25), Kidderminster Union. Mr. F. E. Burcher, C. to G., Kidderminster. March 18.

Laundress (£30), Henley Union. Mr. A. R. Lloyds, C. to G., Henley-on-Thames.

Laundrymaids (£18) (St. George's Guardians. Matron of the Infirmary, Fulham Road, S.W.

Master's Clerk (30s. weekly), Southwark Union. Mr. S. Wood, C. to G., Ufford Street, Blackfriars Road, S.E. March 12.

Master's Clerk (£50), Burnley Union. Mr. J. S. Horn, C. to G., Burnley. March 13.

Messroom Maid (£16), Shoreditch Guardians. Matron of the Infirmary, 204 Hoxton Street, N.

Motor Ambulance Drivers (2) (21s. weekly), Bermondsey Guardians. Mr. E. Pitts Fenton, C. to G., 283 Tooley Street, S.E. March 11.

Needlewoman (£22), Hackney Union. Matron of the Children's Homes, Ongar, Essex.

Nurse (£25), Teesdale Union. Mr. G. Bainbridge, C. to G., Barnard Castle. March 12.

Nurse (£30), Elham Union. Mr. E. Lovick, C. to G., 29 Bouverie Square, Folkestone. March 13.

Nurse (£30), Stockbridge Union. Matron of the Workhouse, Stockbridge.

Nurse (£25), Winchcomb Union. Mr. H. W. Stephens, C. to G., Winchcomb, Glos. March 9.

Nurse (£25), Worksop Union. J. S. Whall, C. to G., 66 Bridge Street, Worksop. March 12.

Nurse (£26), Paddington Guardians. H. F. Aveling, C. to G., 313 Harrow Road, W. March 20.

Nurse-Attendant (£24), Islington Workhouse, St. John's Road, Upper Holloway, N. Matron.

Nurse (£26), Langport Union. E. Q. Louch, C. to G., Bank Chambers, Langport, Som. March 18.

Porter (£25), Ledbury Union. Mr. R. Homes, C. to G., Ledbury. March 11.

Porter and Master's Assistant (£25), Shardlow Union. Mr. J. W. Newbold, C. to G., Becket Street, Derby. March 11.

Probationer (£15), Braintree Union. A. Hills, C. to G., Union Offices, The Workhouse, Bocking, Essex. March 25.

Probationers (£12), Fulham Guardians. E. J. Mott, C. to G., 129 Fulham Palace Road, W.

Relieving Officer, etc. (£80 and fees), Truro Union. Mr. C. Hancock, C. to G., Truro. March 11.

Second Assistant Medical Officer (£130), Leicester Guardians. Mr. H. Mansfield, C. to G., Leicester.

Staff Nurse (£24), Hackney Union, F. R. Coles, C. to G., Sidney Road, Homerton, N.E. March 20.

Staff Nurses (£30), Sheffield Union. Matron, Union Hospital, Fir Vale, Sheffield. March 14.

Superintendents of Home (married couple) (£30 and £25), Cheltenham Union. Mr. J. Meek, C. to G., Cheltenham. March 13.

Ward Sister (£32), Fulham Guardians. E. J. Mott, C. to G., 129 Fulham Palace Road, W. March 12.

Ward Sisters (£30), Brentford Union. Mr. W. Stephens, C. to G., Isleworth, W. March 12.

### Institutional and Administrative Vacancies.

Matron (£60), Beckett Hospital, Barnsley. R. F. Pawsey, Secretary. March 11.

Sister (£35), York Dispensary and Maternity Hospital. Secretary, 4 New Street, York. March 13.

### Municipal Vacancies.

Accounts Clerks (£90 and £78), Hendon U.D.C. Mr. H. Humphris, Clerk to the Council, Hendon, N.W.

Accountant (£130), Prestwich U.D.C. Mr. L. A. Orford, Clerk to the Council, Prestwich. March 16.

Assistant to Water Engineer (£110), Warrington T.C. Mr. J. L. Whittle, Town Clerk, Warrington. March 30.

Charge Nurse (£35), Cuckfield Rural District Council Matron, Dean's Hospital, Hurstpierpoint. March 21.

Charge Nurse (£30), South Shields Rural and Southwick-on-Wear Joint Hospital Board. J. W. Robson, Clerk, Council Offices, Southwick-on-Wear. March 23.

Female Attendants (£19), M.A.B. Medical Superintendent, Leavesden Asylum, King's Langley, Herts.

Gas Works Manager (£150), Liverpool Corporation. The Town Clerk, Liverpool. March 12.

Health Visitor and School Nurse (£80), Windsor T.C. Mr. E. C. Durant, Town Clerk, Windsor. March 9.

Inspectors of Nuisances (2) (£100 each), Smethwick T.C. Mr. W. Shakespeare, Town Clerk, Smethwick. March 16.

Inspector of Nuisances and Surveyor (£75), Llanidloes T.C. Mr. A. Davies, Town Clerk, Llanidloes. March 9.

